# Applications of Virtual Screening in Bioprospecting: Facts, Shifts, and Perspectives to Explore the Chemo-Structural Diversity of Natural Products

**DOI:** 10.3389/fchem.2021.662688

**Published:** 2021-04-29

**Authors:** Kauê Santana, Lidiane Diniz do Nascimento, Anderson Lima e Lima, Vinícius Damasceno, Claudio Nahum, Rodolpho C. Braga, Jerônimo Lameira

**Affiliations:** ^1^Instituto de Biodiversidade, Universidade Federal do Oeste do Pará, Santarém, Brazil; ^2^Laboratório Adolpho Ducke, Coordenação de Botânica, Museu Paraense Emílio Goeldi, Belém, Brazil; ^3^Instituto de Ciências Exatas e Naturais, Universidade Federal do Pará, Belém, Brazil; ^4^InsilicAll Ltda, São Paulo, Brazil; ^5^Instituto de Ciências Biológicas, Universidade Federal do Pará, Belém, Brazil

**Keywords:** machine learning, big data, natural products, bioprospecting, cheminformatics, virtual screening, drug discovery, chemical data

## Abstract

Natural products are continually explored in the development of new bioactive compounds with industrial applications, attracting the attention of scientific research efforts due to their pharmacophore-like structures, pharmacokinetic properties, and unique chemical space. The systematic search for natural sources to obtain valuable molecules to develop products with commercial value and industrial purposes remains the most challenging task in bioprospecting. Virtual screening strategies have innovated the discovery of novel bioactive molecules assessing *in silico* large compound libraries, favoring the analysis of their chemical space, pharmacodynamics, and their pharmacokinetic properties, thus leading to the reduction of financial efforts, infrastructure, and time involved in the process of discovering new chemical entities. Herein, we discuss the computational approaches and methods developed to explore the chemo-structural diversity of natural products, focusing on the main paradigms involved in the discovery and screening of bioactive compounds from natural sources, placing particular emphasis on artificial intelligence, cheminformatics methods, and big data analyses.

**Graphical Abstract:**
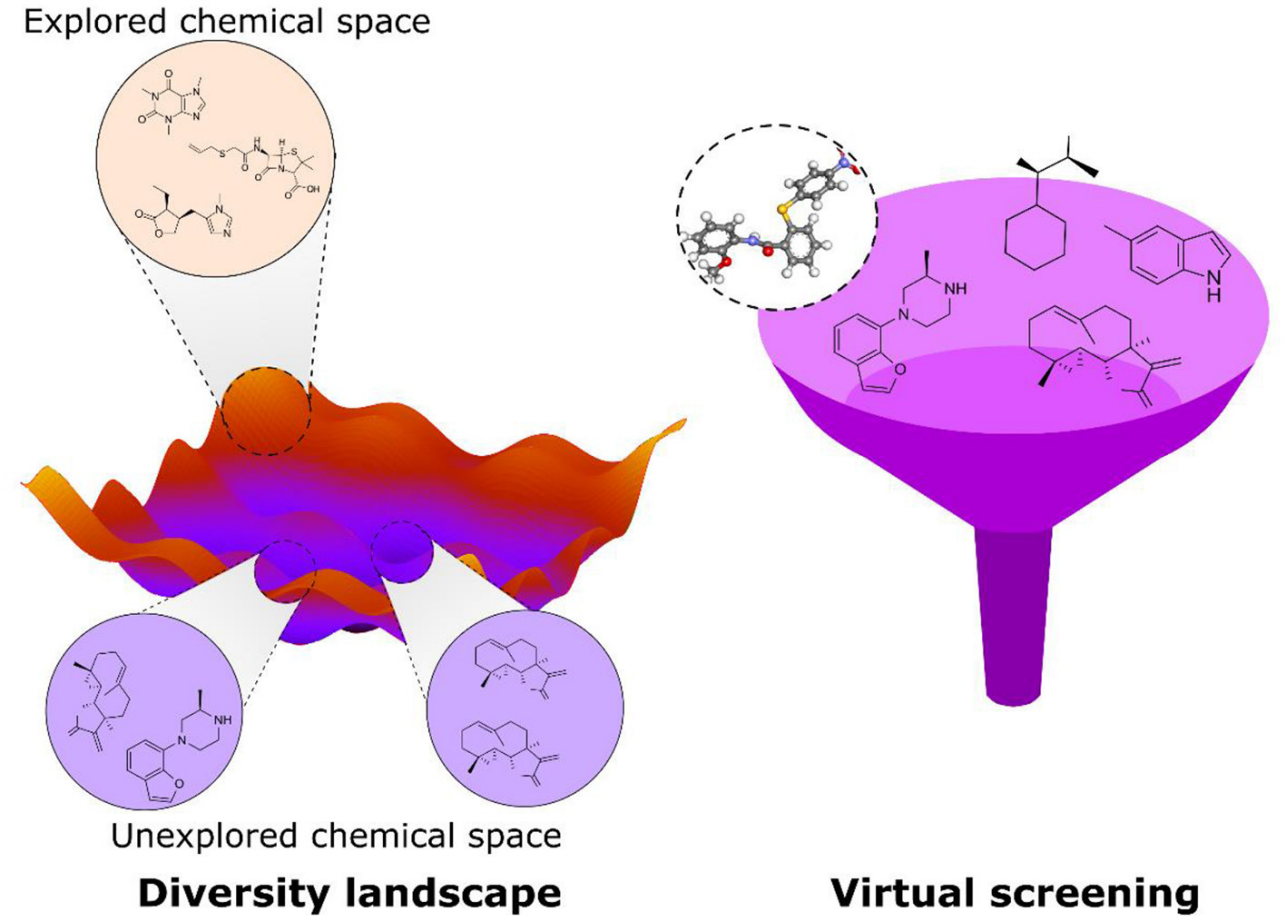
Assessment of the chemo-structural space of natural products using *in silico* tools.

## Natural Products as Sources of Novel Bioactive Compounds and the Paradigms of Their Exploration

The high structural and physicochemical diversity of natural products makes them a valuable source to discover and develop new bioactive compounds with different pharmaceutical, cosmetic, biotechnological, agrochemical, and food applications (Rayan et al., 2017). Success histories of natural product-based drugs have been reported in the pharmaceutical industry and include pilocarpine, quinine, morphine, and artemisinin (Newman and Cragg, 2016; Zhang L. et al., 2020). Natural products represent relevant importance in the discovery and development of new bioinspired bioactive compounds, and more than 50% of the developed drugs approved by the United States Food and Drug Administration (USFDA, 1981–2019) are derived or bioinspired from compounds obtained from natural sources (Newman and Cragg, 2020). Natural products are chemically complex and differ from synthetic compounds in different aspects; as an example, these structures contain a high percentage of oxygen as well as a larger fraction of sp3-hybridized atoms and chiral centers (Lee and Schneider, 2001; Feher and Schmidt, 2003; Rodrigues et al., 2016), and their chemical space is highly diverse, containing different structural scaffolds, when compared with synthetic compound libraries (Chen et al., 2018). Due to their unique features, their structures can provide an innovative solution for the design and synthesis of new bioactive compounds (Kumar et al., 2017; Silva et al., 2019; Bradley et al., 2020; Morais et al., 2020).

The systematic search for natural sources to obtain valuable compounds to develop products with commercial value and industrial purposes remains the most challenging task in bioprospecting (Skirycz et al., 2016; Roumpeka et al., 2017; Cubillos et al., 2019). The traditional approach to discover new bioactive compounds from natural sources includes sequential steps that are obtained from the biological material using ethnological knowledge, extraction, fractionation/isolation, chemical characterization, and, finally, the execution of the biological assays of the isolated or fractionated natural products (Zhang L. et al., 2020). Subsequent analyses include the lead compound optimization using chemical synthesis to perform structural modifications in order to improve their pharmacodynamic and pharmacokinetic properties and to increase their biological activities (Huffman and Shenvi, 2019). In contrast, bioprospecting strategies that use computational tools have been reported as efficient, low-cost, low-labor, and low-time approaches when compared to experimental methods that use solely *in vitro* and *in vivo* assays (Li and Vederas, 2009; Wingert and Camacho, 2018; Trujillo-Correa et al., 2019).

Despite natural products being continually explored in drug development programs, attracting the attention of scientific research efforts due to their pharmacophore-like structures, pharmacokinetic properties, and unique chemical space, the big pharma industry has focused on cutting-edge technologies that combine high-throughput screening and combinatory chemistry methods to obtain and evaluate synthetic compound libraries (Henninot et al., 2018; Batool et al., 2019). This decision is, in part, a consequence of the complex structures of natural products that impose limitations in synthetic routes and due to the time-consuming and laborious process involved in the isolation of a single chemical constituent, which often requires a significant amount of reagents and adequate infrastructure, obtaining low yields of purified target compounds (Huffman and Shenvi, 2019). Based on these limitations, the isolation and the characterization of compounds from natural sources have been indicated only for those with potential applications and desirable biological activities (Olivon et al., 2017). However, it has been suggested that the reduced new chemical entities found by the pharmaceutical industry that reach the final market could be due to the strategic decision to prioritize combinatorial synthetic libraries instead of natural product-based libraries (Over et al., 2013; Rodrigues, 2017). Currently, we are witnessing a resurgence of natural products in the development and research of novel bioactive compounds; besides, some structural scaffolds obtained from different classes of natural products, such as alkaloids, phenylpropanoids, polyketides, and terpenoids, have served as an inspiration to design new drug candidates (Thomford et al., 2018; Davison and Brimble, 2019; Galúcio et al., 2019; Li et al., 2019). Natural products remain inspiring the development of new drugs, cosmetics, and other bioactive compounds for human use (Newman and Cragg, 2020; Atanasov et al., 2021).

Recently, metabolomics and metabolic profiling approaches have explored novel taxonomic groups from the unique environment, providing opportunities for finding novel natural bioactive compounds, and some examples include bacteria (Kleigrewe et al., 2015; Gosse et al., 2019), cnidaria (Santacruz et al., 2020), marine sponge (Abdelhameed et al., 2020), insects (Klupczynska et al., 2020), and fungi (Oppong-Danquah et al., 2018). Special attention has been given to novel chemical entities that originated from marine environments due to their diverse and unique drug-like scaffolds (Shang et al., 2018) and physicochemical properties (Jagannathan, 2019) when compared with natural products of terrestrial origin, which make them a valuable source for exploration by the pharmaceutical and biotechnological industries. Advances in the experimental methods applied in metabolomic approaches coupled with computational methods have been useful to identifying new natural products with plausible biological activities as well as to understanding their molecular mechanisms of action (Atanasov et al., 2021).

Currently, artificial intelligence algorithms (Wolfe et al., 2018; Lima et al., 2020; Stokes et al., 2020) and omics-based technologies (Floros et al., 2016; Huang et al., 2017; Jones and Bunnage, 2017; Merwin et al., 2020) have emerged as approaches to characterize and select interesting chemo-structures with appropriate physicochemical properties and biological activities as well as to prioritize the isolation of natural compounds from biological sources (Chen et al., 2018; Wolfender et al., 2019), which open up new opportunities to explore their industrial applications. Combined with other *in silico* analyses, artificial intelligence and cheminformatics methods can screen a high diversity of chemo-structures isolated from natural sources or deposited in public databases (Chen and Kirchmair, 2020), analyzing their bioactivity, pharmacodynamics, and their pharmacokinetic properties, thus reducing the financial efforts involved in research programs that aim to find new chemical agents (Chen et al., 2018; Al Sharie et al., 2020; Medina-Franco and Saldívar-González, 2020).

In this review, we discuss the computational approaches and methods applied to explore the chemo-structural diversity of natural products, giving particular attention to the main paradigms involved in the discovery and screening of bioactive natural compounds with different industrial applications (e.g., herbicides, insecticides, etc.) that are beyond the discovery of new drugs. Here, we emphasize computational strategies that use artificial intelligence, cheminformatics, and big data analyses that have been developed in the last years. We also explore the limitations and biases of these methods and demonstrate practical applications to evaluate the chemical entities obtained from natural sources aiming at bioprospecting.

## Computational Approaches Applied in the Virtual Screening of Bioactive Compounds

**Figure 1:**
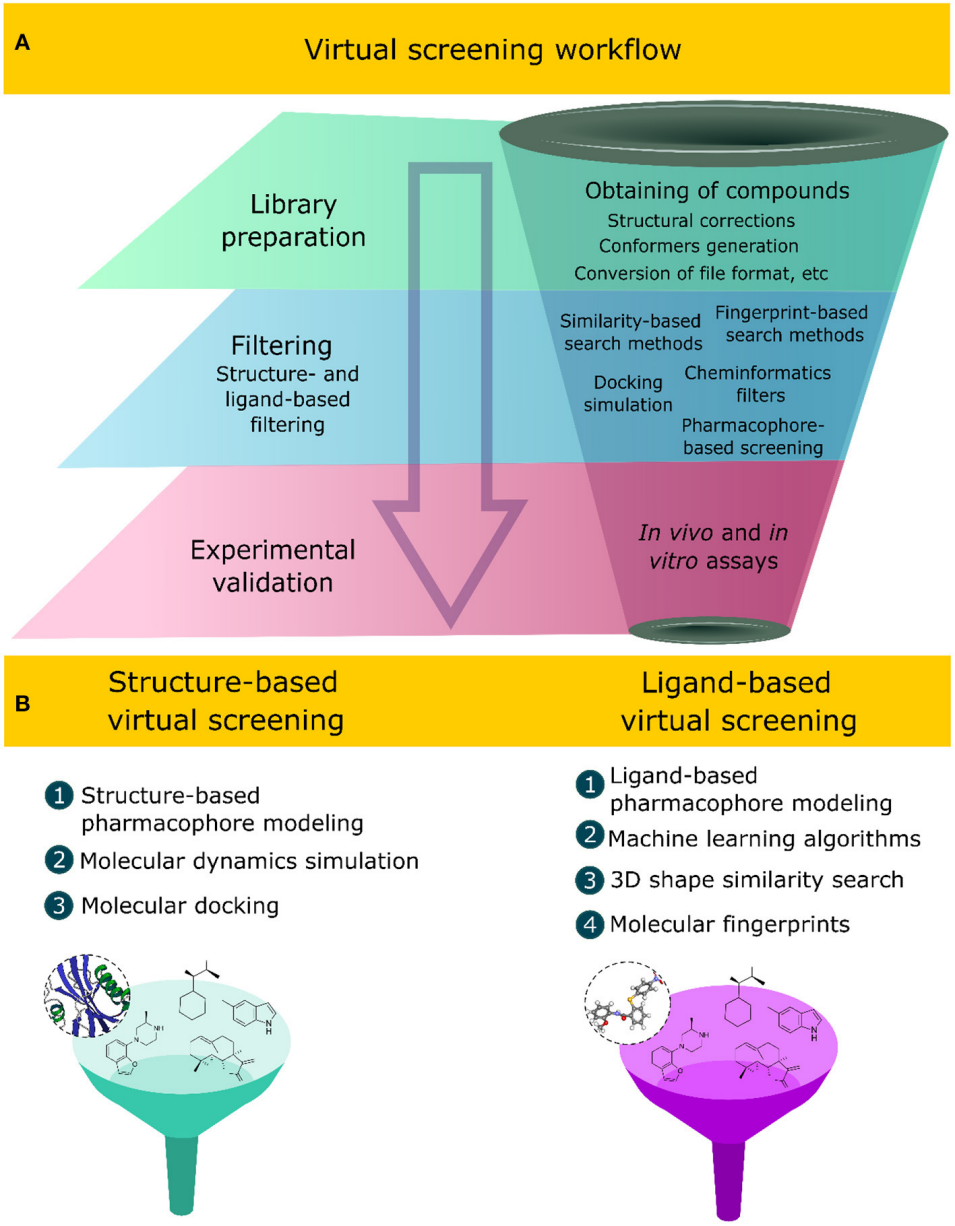
**(A)** Sequential steps applied in virtual screening workflows to select bioactive natural products. **(B)** Ligand- and structure-based virtual screening approaches and some of their associated computational methods.

Virtual screening methods have innovated the discovery of new compounds with specific bioactivity, assessing *in silico* large structural libraries against a bioreceptor or biological system, thus favoring the reduction of financial efforts, infrastructure, and the time involved in the process of discovering new chemo-structures (Macalino et al., 2015). These methods apply sequential and hierarchical steps that aim at filtering and selecting compounds with desirable physicochemical, pharmacokinetic, and pharmacodynamic properties while discarding those that do not fit the desirable characteristics. A virtual screening workflow comprises two main computational tasks (Figure 1A): (1) the first one is the library preparation, which includes, among other computational tasks, obtaining the structures of the compounds, file conversion to readable formats, such as SMILES (simplified molecular-input line entry system), SDF (structure data file), and MOL2 (MDL Molfile) (Saldívar-González et al., 2020), conformer generation, and the correction of stereochemical and valence errors (Ropp et al., 2019); (2) the second one corresponds to the application of computational techniques to filter the desirable compounds (Gimeno et al., 2019). The final step corresponds to experimental validation using *in vitro* and *in vivo* assays, which include enzymatic inhibition assays and/or cell line inhibition (Spyrakis et al., 2019; Ye et al., 2019).

Different computational methods have been developed over the years and implemented in virtual screening strategies (Tomar et al., 2018), applying knowledge of artificial intelligence (Gupta et al., 2013; Yang et al., 2018; Schaduangrat et al., 2019; Shoombuatong et al., 2019; Kong et al., 2020), molecular modeling (Semighini et al., 2011; Rampogu et al., 2018; Da Costa et al., 2019; Jin et al., 2020; Mascarenhas et al., 2020), statistics, and probability (Pire et al., 2015; Daina and Zoete, 2016; Blanco et al., 2018; Madzhidov et al., 2020; Cai et al., 2021). These methods, when combined with experimental approaches, increase the success to finding novel bioactive compounds (Kumar and Zhang, 2015; Coimbra et al., 2020; Gorgulla et al., 2020; Stokes et al., 2020). Two computational approaches are related to the virtual screening of compounds: (1) the ligand-based virtual screening (LBVS) and (2) structure-based virtual screening (SBVS) approaches (Figure 1B). Both computational approaches have been combined in virtual screening strategies that aim to identify novel bioactive compounds against a specific molecular target or a biological system (Da Costa et al., 2019; Galúcio et al., 2019; Wang et al., 2020).

The LBVS approach depends solely on the analyses of the intrinsic characteristics of the compound structure, such as the electronic, topological, physicochemical, and structural properties that are related to its molecular activity using, as a starting point, a set of compounds with experimentally proven biological activity (Hamza et al., 2012; Berenger et al., 2017; Garcia-Hernandez et al., 2019). Computational methods applied in the LBVS approach include structural-, three-dimensional (3D) shape-, and fingerprint-based similarity search methods, cheminformatics filters, machine learning algorithms, ligand-based pharmacophore modeling, and quantitative structure–activity relationship (QSAR) methods (Yan et al., 2016; Tahir et al., 2020). In contrast, the SBVS approach uses, as a starting point, information related to the molecular recognition of the ligand in the bioreceptor structure to design and discover new bioactive compounds. This information includes bioreceptor conformation, the ligand-binding affinity, intermolecular interactions, molecular surface charge, and the composition of the residue of the binding site (Gonczarek et al., 2018; Guedes et al., 2018; Yasuo and Sekijima, 2019; Maia E. H. B. et al., 2020). These methods require the elucidated 3D structure of the receptor and, preferably, in complex with the bioactive compound. The 3D structure informs the structural conformation and molecular binding site of the bioactive ligands. Among the computational methods applied in the SBVS approach, we can cite molecular docking, molecular dynamics simulation, and structure-based pharmacophore modeling (Wang et al., 2020). Currently, virtual screening methods are an integral part of the design and discovery process of new bioactive compounds, and their applications have become popular in the academia and industry (Kar and Roy, 2013).

## Computational Methods Applied in Virtual Screening Approaches

### Cheminformatics Filters (Molecular Filters)

The prediction of the pharmacokinetics and drug-likeness properties of chemical entities represents an important task for the discovery of structures with interesting biological activity (Mignani et al., 2018). In essence, drug-likeness represents a measure of the overall similarity of the analyzed compounds to a chemical space occupied by known drugs (Mignani et al., 2018; Jia et al., 2020).

The prediction of the chemical properties of compounds usually involves the application of a set of simple empirical chemical rules (Gfeller et al., 2014; Lagorce et al., 2015; Daina and Zoete, 2016). Over the years, different cheminformatics filters (also known as molecular filters) have been developed as useful tools to screen structures that have desirable pharmacokinetic and pharmacodynamic properties, low toxicity, and/or low promiscuity/reactivity in inhibition assays, thus guiding the decision-making process in the discovery of new chemical entities with pharmaceutical, cosmetic, agrochemical, and biotechnological interest (Huggins et al., 2011). The most commonly used filters are intended to remove from structural libraries the compounds with low cell membrane permeability or distribution. Among the well-known cheminformatics filters, we can cite those developed by Lipinski (Lipinski et al., 1997), Veber (Veber et al., 2002), and Jeffrey (Jeffrey and Summerfield, 2010). Some structural properties evaluated by these molecular filters predict some pharmacodynamic properties, such as compound promiscuity, i.e., their non-selectivity against a molecular target (Walters and Namchuk, 2003; Lovering, 2013). Some filters are based on the selection of a range of physicochemical and structural properties that are representative of specific pharmacokinetics (e.g., gastrointestinal absorption or penetration into the blood–brain barrier) and pharmacodynamic properties (e.g., specificity or promiscuity to a macromolecular target). These properties are selected using a statistical cutoff (e.g., 90th percentile limit) for each molecular descriptor that is representative to explain the interesting feature of the analyzed compounds (Daina and Zoete, 2016).

Since the first report of the chemical rules elected by Lipinski et al. (1997)—also known as the rule of five (RO5) and Pfizer rules—different chemical extensions to these chemical properties have been developed over the years to better define the “drug-like” features and bioavailability of compounds (Doak et al., 2014). More recently, hybrid methods that combine some counting schemes similar to Lipinski's rules with a set of functional groups identified as reactive, toxic, and problematic moieties have also been developed to eliminate promiscuous structures from the high-throughput screening assays (Walters and Murcko, 2002; Bruns and Watson, 2012). Filters have also been developed to screen fragment-based chemical libraries (rule of three, RO3) (Jhoti et al., 2013). Similar to filters developed for drugs, molecular filters have also been developed to select herbicide-, fungicide-, and insecticide-likeness due to their applications in the agrochemical industry (Tice, 2001; Avram et al., 2014).

Despite these molecular filters having been widely applied in virtual screening approaches to select natural products from large chemo-structural libraries (Thireou et al., 2018; Da Costa et al., 2019; Galúcio et al., 2019), caution must be taken to avoid remotion of the chemo-structures with appropriate bioavailability (Shultz, 2019). Most natural products break some chemical rules applied in molecular filtering; furthermore, some chemical classes of compounds, such as peptides and polyketides (e.g., macrolides), are located beyond the chemical limits determined by the rule of five (beyond the rule of five, bRO5) (Doak et al., 2014; Naylor et al., 2017; Rossi Sebastiano et al., 2018). Contrasting to the drug-likeness, the natural product-likeness concept has been developed to measure the overall molecular diversity of the natural product space, and it has been used as a selection criteria to screen substructures for the prioritization of combinatorial synthesis, aiming at novelty and the easy design of building blocks (Ertl et al., 2008; Jayaseelan et al., 2012). Currently, there are a great variety of cheminformatics programs that calculate these chemical properties that compose the cheminformatics filters, including the open-source programs Osiris DataWarrior [operating system (OS) compatibility: Linux/MS-Windows/Mac OS] (Sander et al., 2015) and RDKit (OS compatibility: Linux/MS-Windows/macOS) (Lovrić et al., 2019), and some commercial solutions, such as Instant JChem (OS compatibility: Linux/MS-Windows/macOS) (Instant JChem 21.4.0, 2021). Similar to these applications, the FAF-Drugs4 web server also predicts some chemical properties to screen structures from large compound libraries using some in-house cheminformatics filters, such as the Drug-Like Soft and Lead-Like Soft that predict compound similarity to drugs and leads, respectively (Miteva et al., 2006). Some databases also offer online tools to evaluate the drug-likeness and natural product-likeness (Sorokina and Steinbeck, 2019; Jia et al., 2020). Table 1 exhibits an overview of the main molecular filters applied to screen natural products from chemical libraries.

**Table 1:** Structural and physicochemical properties present in some cheminformatics filters applied in virtual screening.

	**MW (Da)**	**PSA (A^**2**^)**	**HBA**	**HBD**	**cLog*P*/cLog*D***	**RTB**	**NAR**	**Formal charge**	**References**
Lipinski's rule (RO5)	≤500	–	0–10	0–5	≤5	–	–	–	Lipinski et al., 1997
Ghose's rule	160–480	–	–	–	−0.4 to +5.6	–	20–70	–	Ghose et al., 1999
Oprea's drug-like rule	–	–	2–9	0–2	–	2–8	–	–	Oprea, 2000
Walters	200–500	≤120	0–10	0–5	–	0–8	–	–	Walters and Murcko, 2002
Veber's rule	–	≤ 140	–	–	–	0–10	–	–	Veber et al., 2002
REOS	200–500	–		0–5	−5.0 to 5.0	0–8		−2 to +2	Walters and Namchuk, 2003
Beyond rule of five (bRO5)	≤1,000	<250	<15	≤6	−2 to 10	≤20	–	–	Doak et al., 2014
Congreve's rule (RO3)	<300	–	≤6	≤3	≤3	–	–	–	Congreve et al., 2003
Herbicide-likeness	150–500	–	2–12	<3	≤3.5	<12	–	–	Tice, 2001
Insecticide-likeness	150–500	–	1–18	≤2	0–5	<12	–	–	Tice, 2001
Hao's rule (pesticide-likeness)	≤435	–	≤6	≤2	≤6	≤9	≤17	–	Hao et al., 2011

*MW, molecular weight; PSA, polar surface area; HBD, hydrogen bond donor; HBA, hydrogen bond acceptor; RTB, rotatable bonds; NAR, number of aromatic rings*.

### Molecular Fingerprint-Based Methods

Similarity search methods applied in the screening of natural products are based on the premise that molecules with similar structures have similar biological activities (Cereto-Massagué et al., 2015). These methods have been applied to evaluate natural compound similarities, their bioactivity (Muegge and Mukherjee, 2016), and potential molecular targets (Huang et al., 2018).

**Figure 2:**
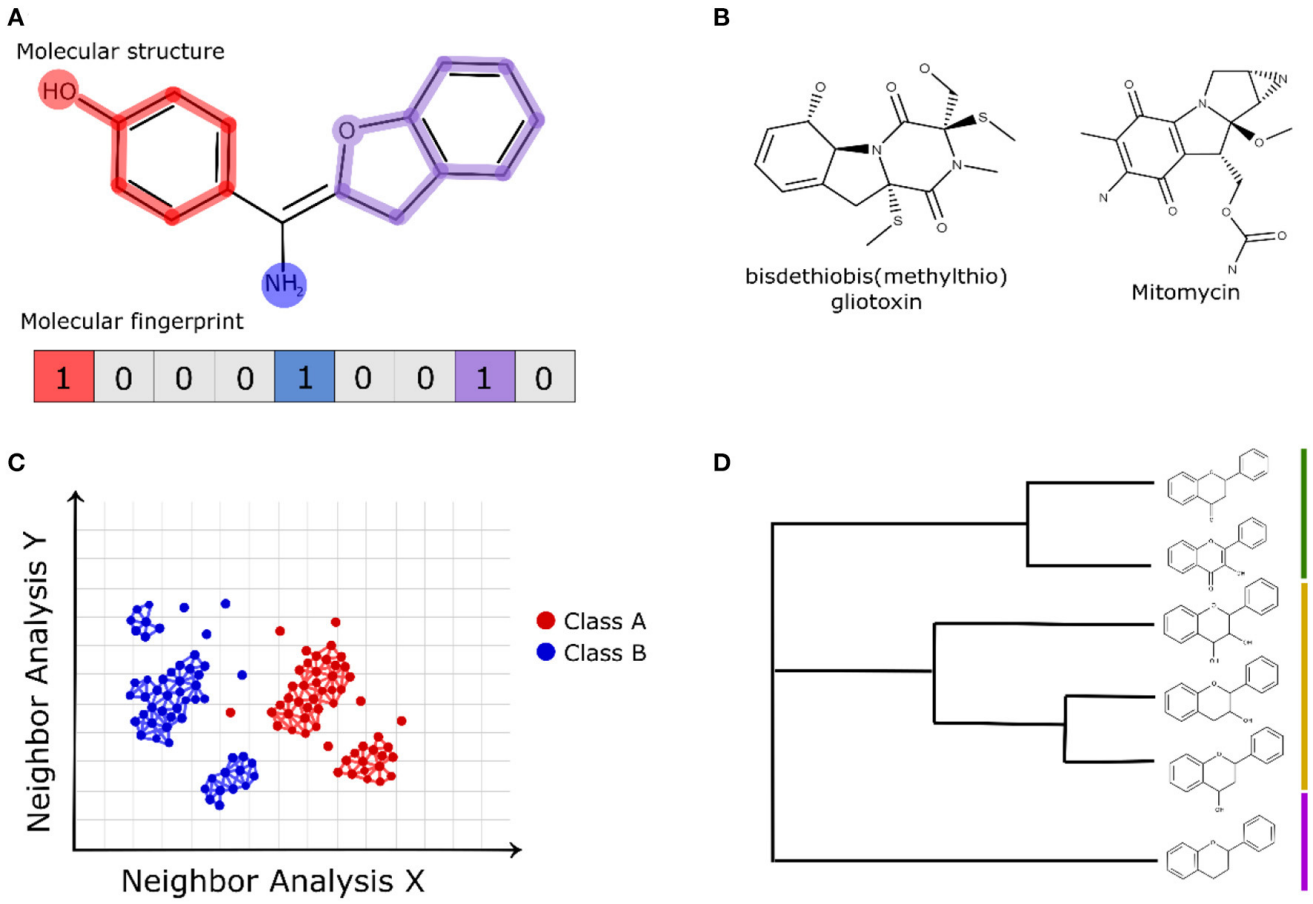
**(A)** Schematic representation of *bits* applied in the molecular fingerprints of chemical structures. **(B)** Fingerprint-based similarity of the natural compound bisdethiobis (methylthio)gliotoxin and the FDA-approved anticancer drug mitomycin (Galúcio et al., 2019). **(C)** Schematic view of the chemical space network and **(D)** hierarchical clustering that apply fingerprint-based descriptors to analyze natural compounds.

Molecular fingerprint-based methods use representations of chemical structures to allow the quantitative assessment of the pairwise similarity of compounds with computationally efficient calculations (Riniker and Landrum, 2013; Bajusz et al., 2015). Molecular fingerprints are binary representations (bits) of a chemical structure in which 1 (present) denotes the existence of a certain molecular feature and 0 (absent) denotes inexistence (Rácz et al., 2018). Figure 2A shows a schematic view of the binary representation of a molecular fingerprint of a compound structure. Molecular fingerprints can vary greatly concerning the applied molecular descriptors, and some of them are based solely on the chemical structure, such as topological distances and the presence/absence of functional groups (Cereto-Massagué et al., 2015). However, some molecular fingerprints use information from pharmacophore models, allowing the comparison of the ligand poses (pharmacophore fingerprints) (Wood et al., 2012). Some molecular fingerprints, such as SMILES fingerprint (SMIfp) (Schwartz et al., 2013), and structural interaction fingerprint (SIFt) (Deng et al., 2004), evaluate structural features related to intermolecular interactions, such as hydrophobic contacts, polar interactions, and hydrogen bond acceptors and donors (interaction fingerprints) (Desaphy et al., 2013). Considering that natural products are chemically complex and structurally different from the synthetic libraries, the analyses of their structures using molecular fingerprints can provide insights, evidencing some structural similarities (see example in Figure 2B) (Gu et al., 2013; Tao et al., 2015; Floros et al., 2016; Galúcio et al., 2019; Chávez-Hernández et al., 2020).

Molecular fingerprints offer a cost-efficient computational calculation to be implemented with other computational approaches. Molecular fingerprints have been widely applied in the representation of chemical space networks to evaluate the structural similarities of natural products (see example in Figure 2C) (Zhang et al., 2015) as well as in hierarchical clustering methods (Figure 2D) (Sánchez-Cruz and Medina-Franco, 2018). In chemical network representations, the nodes (vertices) represent the analyzed compounds and edges of the pairwise fingerprint similarity relationships calculated by a structural metric. The edge drawn between a pair of nodes uses a satisfying threshold criterion for the structural similarity value (e.g., a cutoff = 0.7) between the analyzed compounds (Maggiora and Bajorath, 2014; Kunimoto and Bajorath, 2018). The investigation of the chemical space of natural products is an intelligent way to identify some classes of compounds, their bioactivity, and the structural scaffolds present in known active compounds (Opassi et al., 2018). Due to the high diversity of the derived structures of natural products containing modified functional groups; different strategies have been applied to investigate their chemical space, which include the modeling of hypothetical structural modification (Skinnider et al., 2017) and the application of less restrictive similarity-based cutoffs (Pavadai et al., 2017).

Recently, machine learning algorithms using MACCS keys and Morgan molecular fingerprints have been used to differentiate natural products from synthetic molecules. The authors also used similarity maps to classify natural product substructures according to their similarity to natural or synthetic compounds (Chen et al., 2019). Galúcio et al. (2019) used fingerprint-based similarity to find correspondences between natural products and FDA-approved anticancer drugs, and the authors identified an interesting correspondence (see Figure 2B) between the bisdethiobis(methylthio)gliotoxin obtained from bacterial strain and the FDA-approved anticancer drug mitomycin.

Several programs and web servers have been developed to compute molecular fingerprints, and among them, we can cite ChemDes (web server) (Dong et al., 2015), ChemoPy (open-source Python package) (Cao et al., 2013), PaDEL (open-source Java program) (Yap, 2011), and jCompoundMapper (open-source Java program) (Hinselmann et al., 2011).

### Similarity and Distance Metrics

Structural similarity is a key concept in the discovery of new bioactive compounds from natural sources due to the assumption that similar compounds perform similar molecular activities. Different similarity and distance metrics have been applied to compare molecular fingerprints (Bajusz et al., 2015); some of them are available in cheminformatics tools, such as Konstanz Information Miner (KNIME) (Berthold et al., 2009), PyBel (O'Boyle et al., 2008), the Chemistry Development Kit (CDK) (Willighagen et al., 2017), and RDKit (Lovrić et al., 2019). Similarity metrics could use two-dimensional (2D) or 3D similarities of compounds, but studies have demonstrated that the 2D similarity coefficient neglects some important structural/functional features in the identification of the target compound (Gohlke et al., 2015; Kim et al., 2016).

Several similarities and distance metrics have been applied to compare the pairwise similarities of molecules and their substructures (Bajusz et al., 2015; O'Hagan and Kell, 2016; Rácz et al., 2018). Table 2 exhibits the main similarity coefficients and their dichotomous equations applied to compare molecular fingerprints, where *a* correspond to *on* bits (presence) in structure A, *b* is the number of the *on* bits in structure B, while *c* corresponds to bits that are *on* in both molecular structures. Differently from other similarity metrics, Tversky is an asymmetric coefficient that has two user-defined parameters, α and β. If α is set to 1 and β is set to 0, the Tversky coefficient will measure the substructural similarity between two molecules, where a Tversky value equal to 1 indicates that a given structural moiety is a substructure of the compared compound (Senger, 2009).

**Table 2:** Structural similarity and distance metrics applied in virtual screening.

**Similarity and distance metrics**	**Equations for dichotomous variables**
Cosine coefficient	$S_{\text{A},\text{B}}=c/{\left[ab\right]}^{1/2}$
Dice coefficient	*S*_A, B_ = 2*c*/[*a*+*b*]
Tanimoto coefficient	*S*_A, B_ = *c*/[*a*+*b*−*c*]
Tversky coefficient	*S*_A, B_ = *c*/[α*a*+β*b*−*c*]
Soergel distance	$D_{\text{A},\text{B}}=1-\frac{c}{a+b-c}$
Manhattan distance	*D*_A, B_ = *a* + *b* − 2*c*
Euclidean distance	$D_{\text{A},\text{B}}={\left[a+b-2c\right]}^{1/2}$

Tanimoto has been the most used similarity coefficient in fingerprint-based similarity in virtual screening strategies, and its results have been described, in some cases, as equivalent to other similarity metrics applied to compare two molecules, such as Soergel, Dice, and Cosine, while the similarity measures derived from Euclidean and Manhattan distances have been described as unsatisfactory (Bajusz et al., 2015; Rácz et al., 2018). However, the Tversky coefficient has been indicated to compare moieties of natural products or non-symmetrical scaffolds seeking to identify drug-like similarities (O'Hagan and Kell, 2016). Tanimoto and Tversky coefficient values range from 0 to 1, where values close to 1 correspond to a high similarity between the two analyzed molecules and values close to 0 represent a low similarity (Senger, 2009; Bajusz et al., 2015).

### Ligand-Based and Structure-Based Pharmacophore Modeling

A pharmacophore model consists of a set of chemical groups with a specific 3D arrangement that are involved in biological activity against a specific molecular target (Schaller et al., 2020). The functional characteristics present in a pharmacophore model include hydrogen bond acceptors, hydrogen bond donors, hydrophobic groups, positive or negative ionizable groups, and coordination with metal ions (Vuorinen and Schuster, 2015; Schaller et al., 2020). The binding sites of ligands have physicochemical and spatial restrictions that impose limitations to the non-specific interaction of certain molecules, such as the physicochemical properties of the amino acid residue composition, the volume, and the shape of the cavity. These spatial restrictions dictate the binding mode of the ligands, thus allowing different molecules, even with different structures, to act against a specific bioreceptor due to the presence of the same pharmacophore model (Vuorinen and Schuster, 2015).

Pharmacophore modeling has been extensively applied in virtual screening, lead compound optimization strategies, and *de novo* drug design strategies (Akram et al., 2017; Azminah et al., 2019; Da Costa et al., 2019; El Kerdawy et al., 2019; Jade et al., 2020). Two computational approaches are distinguished in pharmacophore modeling: (1) ligand-based and (2) structure-based approaches. To predict the pharmacophore model, the ligand-based methods use 3D alignment to obtain the chemical information (e.g., shape, functional groups, etc.), shared by a set of active compounds, and select the functional groups that are relevant for the interaction of the ligand with the macromolecular target (Pal et al., 2019). In contrast, the structure-based approach uses the spatial information of the ligand complexed with the molecular target (e.g., ligand *poses*, conformations, etc.); thus, this approach is applied only in the presence of experimentally elucidated structures of the molecular targets (e.g., by X-ray crystallography) complexed with an active ligand (Jiang et al., 2020).

**Figure 3:**
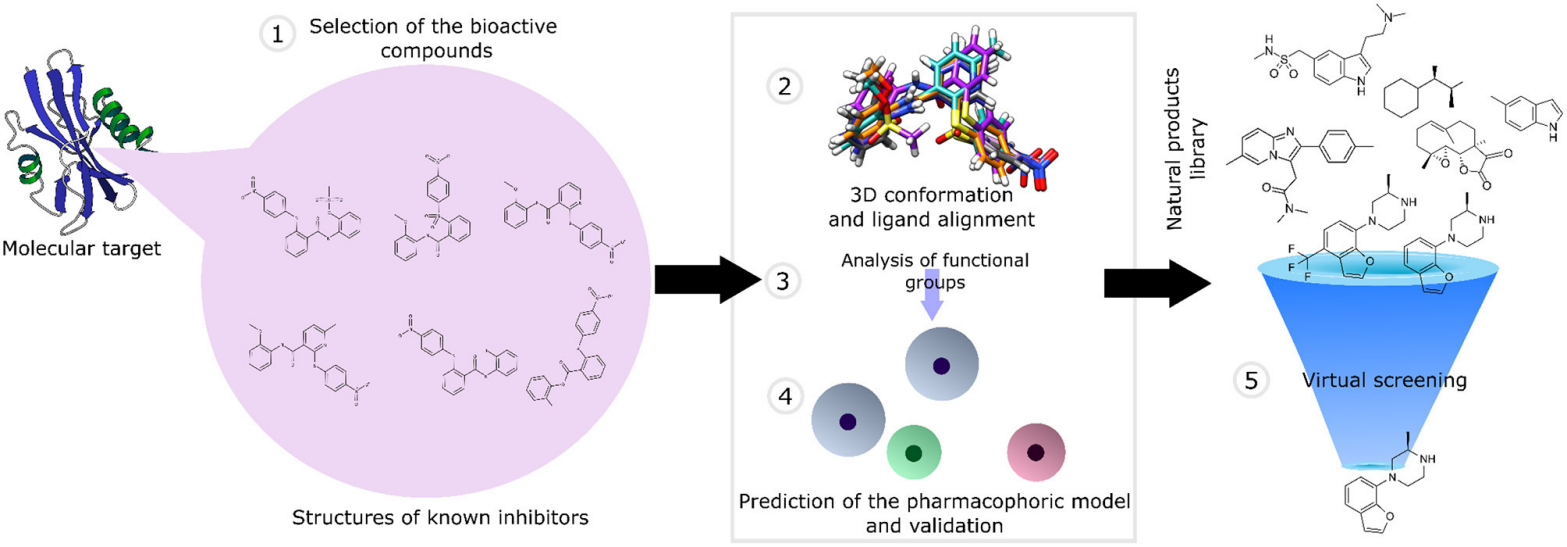
An overview of pharmacophore-based virtual screening applied for natural product libraries.

The ligand-based pharmacophore-based virtual screening comprises different stages: (1) selection of the active compounds validated experimentally; (2) generation of the 3D conformation of the ligands, followed by their structural alignment; (3) identification of the structural characteristics and functional groups involved in molecular recognition; (4) generation and validation of the pharmacophore model using a compound library as a testing dataset; and (5) screening of the natural product library (Figure 3).

In ligand-based pharmacophore modeling, the pharmacophore model is generated using a 3D alignment of the conformers of a set of bioactive compounds (training dataset). Then, active (true-positive compounds or hits) and inactive compounds (false-positive compounds or decoys) are used as a testing dataset to validate the pharmacophore model (Shahin et al., 2016; Pal et al., 2019). It is important to note that, despite the choice of strict pharmacophore models leading to the selection of compounds with better activities against the molecular target, it also could reduce the structural diversity of the analyzed natural products. In contrast, the choice of less restrictive models could retrieve a larger number of false-positive compounds (Schaller et al., 2020).

Pharmacophore modeling methods could be divided into two scoring function methods to predict the fitness of the analyzed compounds to the predicted pharmacophore models: the root of the mean square deviation (RMSD)-based and the overlay-based scoring function (Sanders et al., 2012). In RMSD-based methods, the distances between the functional groups of the compounds to the center of pharmacophore features are used to assess the fitness of the compounds concerning the predicted pharmacophore model. In contrast, the overlay-based methods use the *radii* of the functional groups and/or atoms to estimate the functional similarity of the structures with the pharmacophore model (Vuorinen and Schuster, 2015). Pharmacophore-based methods that apply RMSD-based scoring functions are better at predicting the ligand poses than the overlay-based scoring functions (Sanders et al., 2012). Nevertheless, the ratio of correctly predicted poses vs. incorrectly predicted poses is better obtained using overlay-based scoring functions (Sanders et al., 2012). Regarding structure-based pharmacophore modeling, the use of experimental structures to build the models must prioritize some structural features obtained from both methods; as an example, it has been demonstrated that a higher flexibility obtained in structures elucidated by nuclear magnetic resonance (NMR) spectroscopy helps to focus the models on the most essential interactions with the receptor due to the presence of structural flexibility of the complexes evidenced by the method. On the other hand, models obtained by X-ray crystallography had more pharmacophore elements compared to those obtained by NMR spectroscopy (Ghanakota and Carlson, 2017).

Pharmacophoric screening has been applied to screen compounds with cosmetic purposes using essential oils (Santana et al., 2018; Da Costa et al., 2019). Essential oils contain diverse classes of volatile and low-molecular-weight compounds with a broad spectrum of biological activities (Do Nascimento et al., 2020), and due to their reported repellent activities against mosquitos, these compounds have been investigated in virtual screening strategies (Santana et al., 2018; Thireou et al., 2018). Recently, a study performed an *in silico* analysis of 1,633 compounds from the essential oils of 71 botanical families by combining a structural similarity-based search method (ligand-based virtual screening) with a pharmacophore-based virtual screening (structure-based strategy). The authors used, as a reference, the structure of *N,N*-diethyl-*meta*-toluamide (DEET) complexed to the odorant-binding protein of *Anopheles gambiae*, and they found seven natural volatile compounds with potential repellent activity against mosquitos, such as *p*-cymen-8-yl, thymol acetate, carvacryl acetate, thymyl isovalerate, and *p*-anisyl hexanoate (Da Costa et al., 2019).

Currently, different programs generate pharmacophore models, differing in the algorithm applied to evaluate the conformational ligand flexibility as well as to perform the structural alignment. Some commercial programs applied to pharmacophore prediction include LigandScout (Wolber and Langer, 2005) and Molecular Operating Environment (MOE) (Molecular Operating Environment, 2019). Both programs apply ligand- and structure-based pharmacophore modeling and are compatible with the most used operating systems. Some open-source programs that use ligand-based pharmacophore prediction include Pharmer (https://sourceforge.net/projects/pharmer/) (Koes and Camacho, 2011) and Align-it (previously named Pharao; OS compatibility: OS X) (Taminau et al., 2008). Free-access web servers have also been developed to screen compounds using the structure-based pharmacophore approaches, such as Pharmit (http://pharmit.csb.pitt.edu/) (Sunseri and Koes, 2016) and PharmMapper (http://www.lilab-ecust.cn/pharmmapper/) (Liu et al., 2010).

### 3D Shape-Similarity Search Methods

The molecular shape acquired by a ligand is crucial to defining its affinity and selectivity against the protein binding site (Kortagere et al., 2009). Based on this assumption, the 3D shape-similarity search methods assume the premise that two compounds could be recognized by the same bioreceptor and then modulate their activity (Koes and Camacho, 2014; Kumar and Zhang, 2018). Shape-similarity methods can screen vast compound libraries against a reference ligand with known bioactivity (Ai et al., 2014; Koes and Camacho, 2014).

These methods are subdivided into two categories: (1) alignment-free methods that are usually computationally faster because they do not require overlapping the molecules or evaluating properties related to the surface (Seddon et al., 2019) and (2) alignment-based methods that are computationally costly since these methods superimpose molecular shapes and analyze surface properties, such as polarity and hydrophobicity (Fontaine et al., 2007; Kumar and Zhang, 2018). Different methods have been used in the representation of the 3D molecular shape of the ligands, such as Gaussian overlay-based methods (Cai et al., 2013), atomic distance-based methods (Ballester et al., 2009; Ballester, 2011; Bonanno and Ebejer, 2020), and surface-based methods (Karaboga et al., 2013; Cleves et al., 2019). The recognized molecular shapes are transformed into the 3D molecular fingerprints that are then compared using similarities or distance indexes, such as Tanimoto, Dice, and Tversky coefficients (Shin et al., 2015). Due to the complex structure of natural products, the identification of their molecular targets has been challenging even using computational tools; however, the 3D shape-based similarity search methods have emerged as an efficient strategy to predict the macromolecular targets of these compounds (Shin et al., 2015; Chen et al., 2020). Web servers that apply shape-similarity search methods include the SHAFTS (Liu et al., 2011) and USR-VS (Li et al., 2016). Some installable open-source programs include Shape-it (OS compatibility: Linux) (Grant et al., 1996), gWEGA (Yan et al., 2014), and OptiPharm (Puertas-Martín et al., 2019). Some commercial solutions include Shape TK (OS compatibility: Linux/MS-Windows/macOS) (Software O Scientific, 2008).

**Figure 4:**
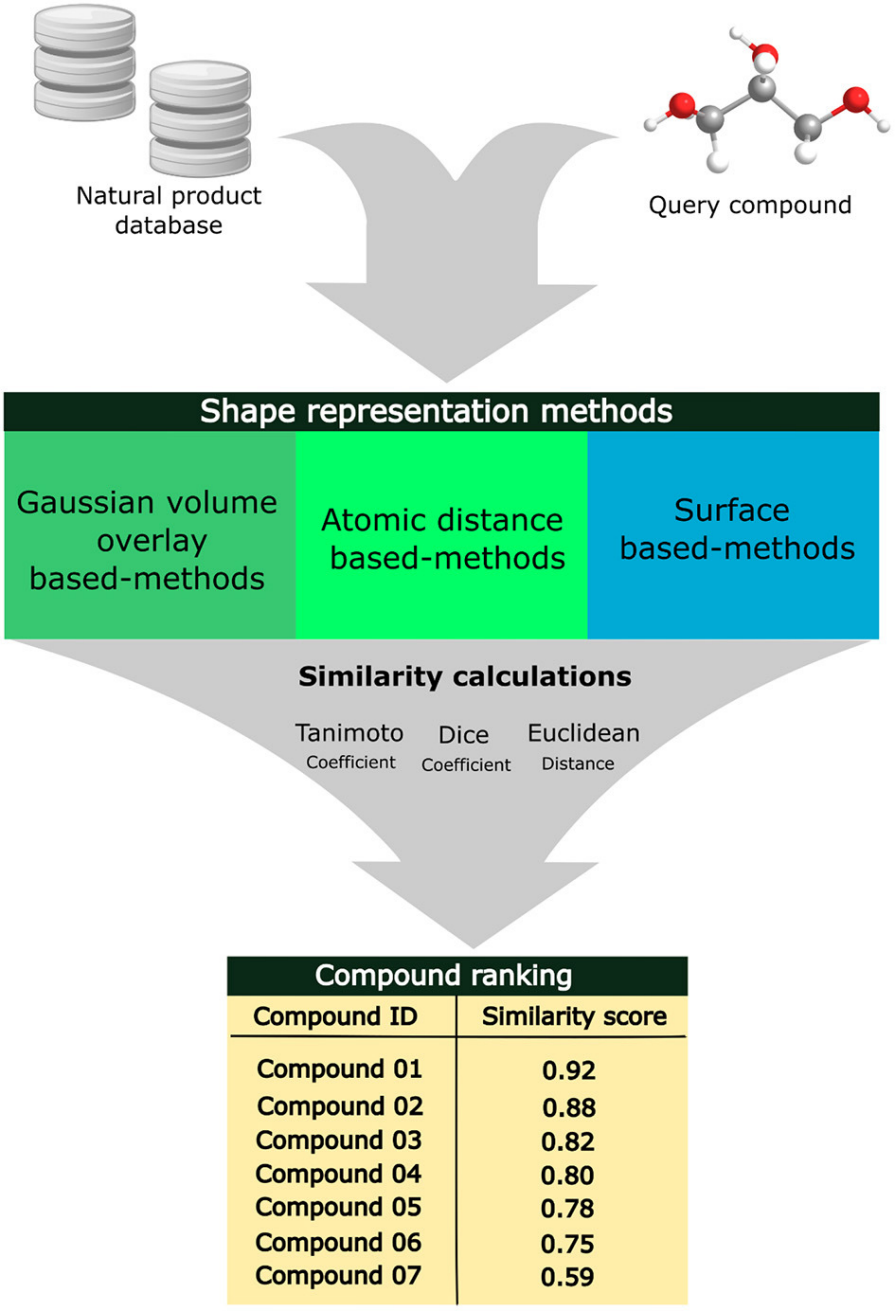
Applications of alignment-based 3D shape-similarity search methods to identify compounds with similar molecular shapes.

Shape-based similarity methods have been used in virtual screening workflows alone or combined with different computational techniques (Pavadai et al., 2017; Thireou et al., 2018). Pavadai et al. applied shape-based and fingerprint-based similarity search against natural product libraries to find new steroid-like natural products as antiplasmodial agents using, as a search key, fusidic acid. The hit compounds were filtered based on the predicted partition coefficient, log*P*, and the authors identified nine new compounds that inhibited parasite growth with IC_50_ values of <20 μM (Pavadai et al., 2017). Figure 4 exhibits an overview of the 3D shape-similarity search methods applied to identify compounds in chemical libraries with similar molecular shapes despite their different structures.

### Machine Learning Algorithms

Machine learning (ML) is the computational practice of using intelligent algorithms to learn and make decisions in order to solve problems related to an amount of data. Artificial Intelligence has made important progress toward the acceleration of research and development of novel bioactive natural compounds with industrial applications. This approach has been widely applied in different steps related to the virtual screening strategies, for example to predict some pharmacokinetic properties (Wei et al., 2017; Qiang et al., 2018) [e.g., penetration of compounds into the blood–brain barrier (Zhang et al., 2017; Dai et al., 2021) and cell membrane (Wei et al., 2017; Wolfe et al., 2018)], compounds' side effects (Dimitri and Lió, 2017), their toxicity (Mayr et al., 2016; Pu et al., 2019; Zheng et al., 2020), molecular targets (Wang et al., 2013; Jeon et al., 2014), and their bioactivity (Li and Huang, 2012; Schaduangrat et al., 2019; Shoombuatong et al., 2019) [e.g., anti-tuberculosis (Gomes et al., 2017; Maia S. M. et al., 2020), anticancer (Charoenkwan et al., 2021), and insecticidal activities (Soares Rodrigues et al., 2021)] as well as to identify the pan-assay interference compounds (PAINS), i.e., highly reactive and promiscuous molecules that are often false positives in high-throughput screening assays (Jasial et al., 2018). In some cases, the ML algorithms have been reported with superior efficiency and, thus, are more suitable to predict hit compounds from chemical libraries than are the traditional QSAR methods (Tsou et al., 2020).

ML algorithms are trained using a large number of data that are used as a benchmark to accomplish a particular computational problem (Vamathevan et al., 2019). The main aim of an ML framework in virtual screening strategies is to generalize the results obtained from the training dataset to better evaluate the test dataset and, then, make the decision (Sieg et al., 2019; Vamathevan et al., 2019). ML algorithms applied in the LBVS approach aim to predict the bioactivity or pharmacodynamic/pharmacokinetic properties of molecules based on their similarity to known actives. Therefore, to evaluate the similarity of the molecules, these algorithms use, as datasets, molecular descriptors calculated from the compound structures (Li and Huang, 2012; Challa et al., 2020) using different molecular modeling and cheminformatics toolkits, such as RDKit (Lovrić et al., 2019) and CDK (Willighagen et al., 2017). Some chemo-structural and bioactivity information deposited in public databases, as well as experimental results, have also been used to train these algorithms (Martínez-Treviño et al., 2020). Molecular descriptors applied to evaluate the similarity of molecules include the physicochemical [cLog*P*, topological polar surface area (tPSA), molecular weight, etc.] and structural properties (rotatable bonds, aromatic rings, etc.) (Lo et al., 2018), molecular fingerprints (Zhang et al., 2018), functional groups, molecular shape (Bonanno and Ebejer, 2020), and pharmacophores (Sato et al., 2010); in the case of proteins and peptides, some molecular descriptors include amino acid sequence composition (Wei et al., 2017; Manavalan et al., 2018; Qiang et al., 2018). The choice of the molecular representation and the type of molecular descriptor determine the efficiency and the interpretability of the final results obtained by the ML algorithms (David et al., 2020; Jiménez-Luna et al., 2020). In structure-based strategies, ML algorithms have been used in scoring the functions of molecular docking methods, seeking rank compound libraries based on their predicted affinity against a molecular target, and discriminating between hits and decoy compounds. To reach these results, the ML algorithms are trained using the binding affinities of active molecules against protein targets (Wójcikowski et al., 2017; Li et al., 2020). Different open-source programs have been applied to develop machine learning models [e.g., scikit-learn (Pedregosa et al., 2011) and SciPy (Virtanen et al., 2020), both Python modules] and pipelines [e.g., KNIME (Berthold et al., 2009), a data analytics platform].

**Figure 5:**
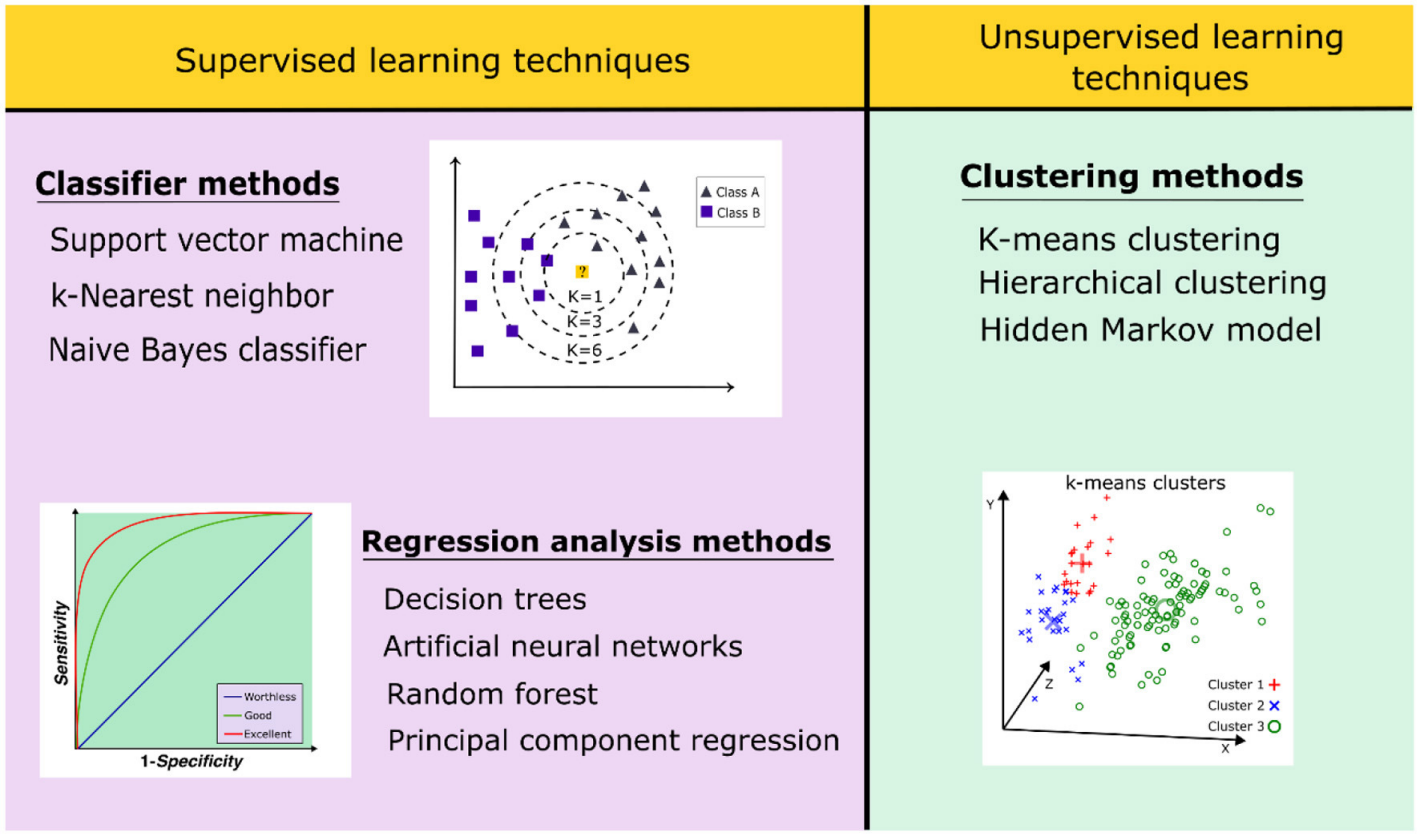
Classification of supervised and unsupervised learning techniques applied in virtual screening strategies.

ML algorithms are classified into supervised and unsupervised learning (Figure 5). Supervised ML algorithms require a retrospective validation using a dataset of active and inactive compounds to better select the methods that are suitable to differentiate the bioactive molecules (Sieg et al., 2019). Supervised learning techniques are divided into two subgroups: (1) regression analysis and (2) classifier methods. The first one includes decision trees, artificial neural networks, support vector machines, and random forest methods. In contrast, the unsupervised algorithms recognize patterns in the dataset of compounds without the presence of inactive ones, thus trying to organize the data in a logical form. These methods have been used for exploratory analyses using clustering data (Patel et al., 2020). Unsupervised algorithms include clustering methods, such as the hidden Markov model, hierarchical clustering, and *k*-means (Vamathevan et al., 2019).

**Figure 6:**
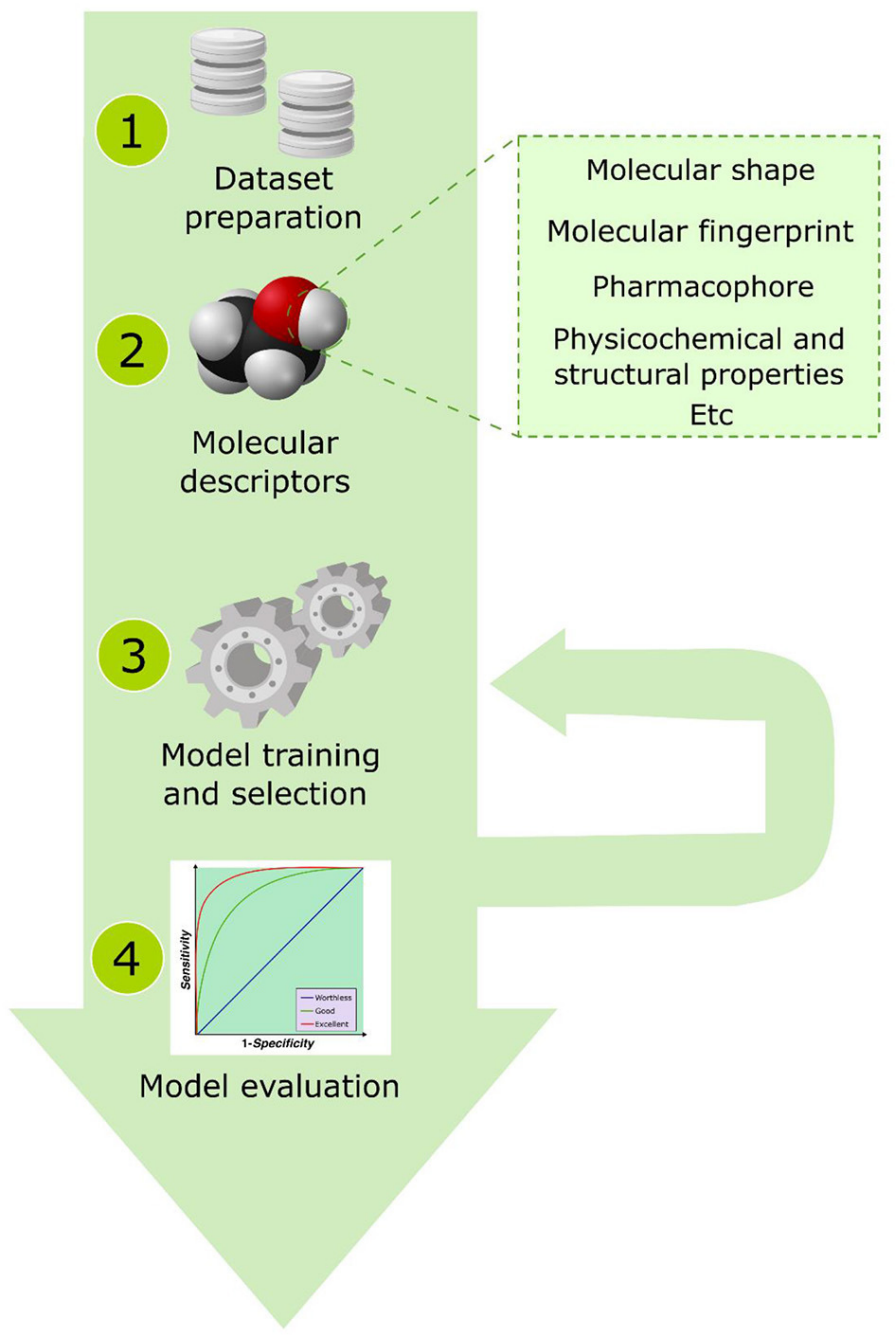
A general overview of the computational steps involved in obtaining a validated supervised machine learning algorithm.

Supervised ML algorithms have been widely applied to discover new bioactive natural products (Bilsland et al., 2015; Galúcio et al., 2019; Grisoni et al., 2019; Schaduangrat et al., 2019). Figure 6 exhibits a general overview of the computational steps involved in obtaining a validated supervised ML algorithm to predict the bioactivity of natural products. The first step to modeling a machine learning algorithm involves the preparation of a molecule dataset, i.e., obtaining the molecular structures/sequences that will be used in the algorithm using online databases, literature, or experimental data. This step also includes the correction of possible stereochemical and valence errors present in the molecular structures as well as the correction and conversions of the files to readable formats recognized by the cheminformatics programs. Then, the molecular properties are calculated using molecular modeling and cheminformatics toolboxes, extracted from online databases, or obtained from experimental results, then these descriptors are evaluated to compose the features of the ML model. Currently, different online databases have been developed with information regarding the structural and physicochemical properties of the molecular structure of natural products that could be used in the feature composition (Dunkel et al., 2006; Pilon et al., 2017; Pilón-Jiménez et al., 2019; Sorokina and Steinbeck, 2019). In this step, some statistical methods are applied to select the features, such as Kendall correlation, analysis of variance (ANOVA), and Spearman's test. Finally, the ML model is evaluated regarding its performance to discriminate the true and positive bioactive compounds. Several metrics have been applied to evaluate these models, such as the receiver operating characteristic (ROC) curve, enrichment factors, and mean squared error (*R*^2^) applied for linear regression methods. We do not intend to extend the discussion about the application and the choice of the most adequate method to select the feature composition or to evaluate ML models; thus, we recommend the readers to consult previous reviews (Hossin and Sulaiman, 2015; Rácz et al., 2019). In the present sessions, we will discuss the functioning of some ML algorithms most applied in virtual screening strategies focusing on the *k*-nearest neighbor, decision tree, random forest, artificial, and neural network.

**Figure 7:**
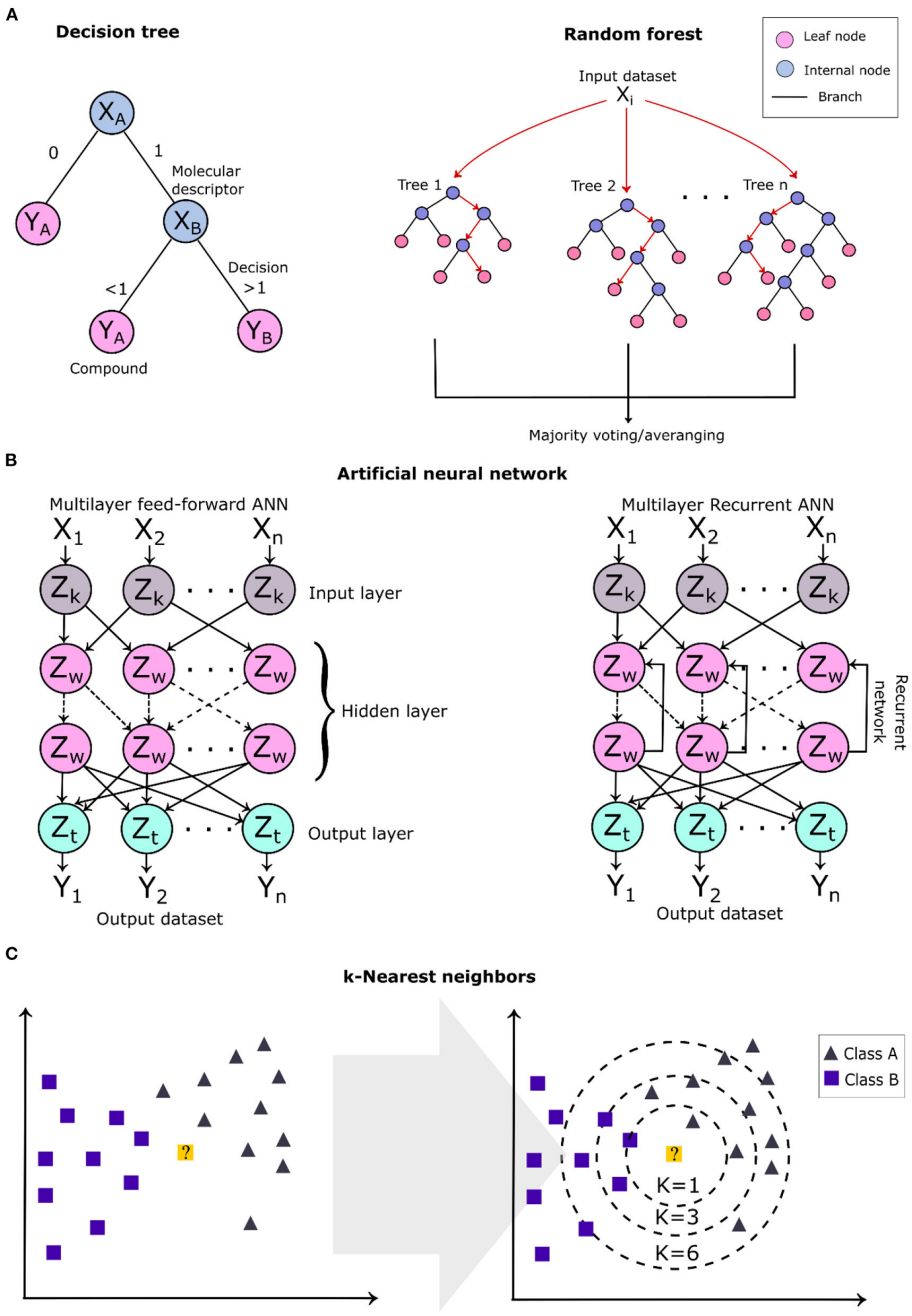
Schematic overview of some of the machine learning algorithms applied in virtual screening. **(A)** Two-dimensional (2D) diagram of a single root tree of a decision tree algorithm and the general architecture of a random forest. **(B)** The architecture of a multilayer feed-forward and recursive artificial neural network. *Z*_w_ refers to neurons of the hidden layers (internal); *Z*_k_ and *Z*_t_, to the neurons of the input and output layers, respectively. **(C)** *k*-Nearest neighbor algorithm showing the learning technique to classify a new data represented by the 2D *yellow point*, which is classified as belonging to class A (*gray triangles*).

Decision tree algorithms are a supervised learning technique and their construction model is based on two steps: (1) selection of the features and (2) the building of the decision trees. This method is commonly represented by a tree, where the internal nodes represent the selected features (molecular descriptors), the branches represent the testing results of the molecule (decision criteria), and the leaf nodes represent the molecules (molecular structure) (Figure 7A). Compounds are classified based on the leaf nodes that are reached through a series of algorithm decisions (branches). Decision tree (DT) models are constructed focusing on the selection of the best test conditions to expand the extremities of the tree. Some test metrics, such as the information–gain ratio and entropy, are applied to select the best test classification for the algorithm (Lavecchia, 2015). Decision trees have been applied in different virtual screenings of natural products to predict their bioactivity and drug-likeness (Pereira et al., 2015; Wang et al., 2019). Random forest is an ensemble learning technique considered an improvement of the decision tree algorithms to correct the overfitting in the training set (Svetnik et al., 2003). Random forest algorithms generate a model composed of several randomly sampled decision trees from the original dataset obtaining its random features. Random forest models have been applied in virtual screening pipelines to predict compound drug-likeness, bioactivity (Svetnik et al., 2003; Zoffmann et al., 2019), and the pharmacokinetic profile (Dong et al., 2018).

Artificial neural networks are the most studied learning techniques with widely diverse applications in the investigation of a compound's bioactivity (Lata et al., 2007; Liu et al., 2019, 2020; Stokes et al., 2020). Methods that apply neural networks mimic brain functioning and structure, building a model that reaches a decision based on previous experiences obtained from the training dataset (Jing et al., 2018). The architecture of an artificial neural network model comprises several units, named neurons which are connected to form a network arranged in different layers. Depending upon their position in the network, these layers are classified as output layers, input layers (external), and hidden layers (internal) (Zhang R. et al., 2020). A multilayer feed-forward neural network contains neurons connected only to those located in the following layers (Figure 7B), and this class is included in radial basis function networks, multilayer perceptrons, and self-organizing maps (Kohonen maps) (Lavecchia, 2015). In contrast, the recurrent neural networks contain feedbacks between the layers, i.e., interconnections between neurons from the same and consecutive layers; thus, their outputs are determined by the previous outputs and the current inputs (Figure 7B), which form a “memory” during the learning process.

The *k*-nearest neighbor is instance-based learning and is one of the simplest and intuitive ML algorithms applied to classify and rank compounds based on the nearest training examples present in the chemical space (analyzed feature composition) (Kauffman and Jurs, 2001; Medina-Franco et al., 2005). The algorithm compares the molecular descriptors of the query molecule with *k*-neighbors that have the smallest distance (*k*-value), where the *k*-value corresponds to the number of closest neighbors (a positive integer) and classifies them by majority votes of their closest neighbors (Figure 7C). The number of neighbors is the most important parameter for the model, deciding its complexity. *k*-nearest neighbor is a classifier algorithm; thus, irrelevant features can lead to disturbances in the compound classification. It is indicated to first preprocess the molecular descriptors to remove the irrelevant or the most correlated ones.

Despite the majority of the computational screening approaches using ML algorithms lacking experimental validations, we have some interesting successful studies that aimed to find and characterize novel natural products with experimentally validated biological activity (Rupp et al., 2010; Zhang et al., 2017; Nocedo-Mena et al., 2019; Patsilinakos et al., 2019; Lee et al., 2020; Liu et al., 2020). Recently, Reher et al. reported on the SMART 2.0, an NMR-based machine learning tool designed for the discovery and characterization of natural products. The tool was successfully applied to investigate the environmental extract of *Symploca* sp., a filamentous marine cyanobacterium, leading to the isolation and identification of a new chimeric macrolide named symplocolide A. The molecular structure of this novel natural product was confirmed by 1D/2D NMR and tandem liquid chromatography mass spectrometry (LC-MS^2^) analysis (Reher et al., 2020). Similarly, Lee et al. applied SMART 2.0 to prioritize the isolation and characterization of sesquiterpene lactones from the *Eupatorium fortune* plant. The isolated natural compounds were experimentally tested against five cancer cell lines and exhibited cytotoxic activities (Lee et al., 2020).

ML algorithms have been successfully applied to predict the bioactivity of compounds. Recently, Nocedo-Mena et al. (2019) combined machine learning, perturbation theory, and information fusion techniques to investigate the antibacterial activity of terpenes from the *Cissus incisa* plant, and the authors found that phytol and α-amyrin showed minimum inhibitory concentrations equal to 100 μg/ml against the carbapenem-resistant *Acinetobacter baumannii* and the vancomycin-resistant *Enterococcus faecium*. In another study, Liu et al. applied deep learning algorithms to find natural products with anti-osteoporosis activity. The selected hits successfully suppressed the osteoclastogenesis-related genes *Rank, Tracp, Ctsk*, and *Nfatc1 in vitro* (Liu et al., 2020). Some studies have also reported experimental validations of ML models to predict pharmacokinetic properties. Zhang et al. used a hybrid ML algorithm using support vector machine, probabilistic neural network, naive Bayes classifier, and random forest models combined with *in vitro* assays to predict the blood–brain barrier penetration of natural compounds from the Traditional Chinese Medicine database (TCM_DB_). The authors found an overall accuracy for experimental validation around 81% (Zhang et al., 2017).

## Biases and Limitations of Virtual Screening Methods

Virtual screening approaches have been predictive, useful, and cost-effective in identifying novel bioactive compounds when compared with the traditional methods applied solely. However, despite their well-known success, these methods have limitations and their models are prone to biases (Sieg et al., 2019; Slater and Kontoyianni, 2019). It has been demonstrated that the presence of stereochemical and valence errors in the chemical data libraries could also induce investigators to choose unfeasible compounds (Williams and Ekins, 2011; Williams et al., 2012).

Biases, in essence, correspond to distortions from the true underlying relationship between the investigated objects. The investigation of the chemo-structural diversity of natural products and their bioactivity using similarity-based search methods is biased because it considers an assumption that the discovery of novel active compounds must consider the similarity of known active ones (Sieg et al., 2019). This assumption is susceptible to drive the decision-making process to erroneous directions and can reduce the structural diversity of new chemo-structures. Combining low time-consuming computational simulations and more realistic results also remains a challenge for some 3D similarity-based search algorithms, which, in general, require superimposing many conformation pairs of compounds from large chemical libraries, thus requiring high-performance computing (Yan et al., 2016).

Despite the chemical space being considered infinite, the pharmacological space of bioactive compounds of the “druggable human genome” is limited, and its exploration remains a difficult task even from a computational point of view (Opassi et al., 2018). This assumption has been proven to be true for other classes of bioactive compounds with industrial applications, such as pesticides and herbicides (Avram et al., 2014). Therefore, the exclusion of some compounds during the filtering process is comprehensive, but can also reduce the investigation of new chemical entities with specific bioactivity.

In pharmacophore-based virtual screening, the selection of inappropriate models, or very restricted ones, could eliminate an interesting structural diversity of natural compounds. However, the choice of less restrictive models could retrieve a larger number of false-positive compounds (Lans et al., 2020; Schaller et al., 2020). Based on these biases, a balanced choice between strict and loose criteria to select the pharmacophore model to filter natural products could be decided by prioritizing pharmacophore moieties better associated with a higher compound activity; thus, the information obtained from structure–activity analyses might be useful to decide on the most appropriate pharmacophore model to screen natural products (Qing et al., 2014). Regarding the limitation of ligand-based pharmacophore modeling methods, it has been reported that their dependence on structurally similar compounds reduces their application since compounds with high structural dissimilarities may not share the same binding mode (Schaller et al., 2020). Furthermore, few ligand-based methods consider the conformational flexibility of the macromolecular receptor in the determination of the pharmacophore model (Lans et al., 2020). In molecular docking, for example, the elimination of compounds with poor fitness could be biased due to the choice of wrong or inappropriate scoring functions, i.e., those that contain chemical information that contradicts the physical reality or that were not calibrated for the class of investigated molecules (Luo et al., 2017).

Supervised machine learning algorithms are also prone to biases, which can lead to a misleading interpretation of the final results obtained for chemical data libraries. It has been demonstrated that highly correlated training and testing datasets, i.e., containing chemical data too closely similar (e.g., same molecular scaffold with a high frequency between the datasets), could limit the applicability of the machine learning model, reaching false accuracies in its predictiveness (Wallach and Heifets, 2018; Sieg et al., 2019). Therefore, low training errors are insufficient to justify the choice of a machine learning model since the satisfactory predictive performance could be due to redundancy between the training and testing datasets rather than accuracy (Wallach and Heifets, 2018). It has also been demonstrated that some biased machine learning models could be obtained using a training dataset composed of active molecules that are easily differentiated from inactive ones by coarse properties, such as cLog*P*, the number of HBA, and molecular weight (Ripphausen et al., 2011). Based on these biases of machine learning models, it is necessary to investigate whether chemical data benchmarks contain design flaws that might lead to optimistic performances that are distorted from the chemical reality. Some computational methods have been developed to avoid overfitting in chemical datasets. Wallach and Heifets (2018) developed the asymmetric validation embedding (AVE) bias using Python language to predict the performance across common benchmarks and standard machine learning algorithms, and Ripphausen et al. (2011) developed a public compound database, named REPROVIS-DB, that contains information from successful ligand-based virtual screening strategies including experimentally confirmed hits, reference compounds, screening databases, and selection criteria.

## Natural Products Databases Applied in Virtual Screening

The development of computational approaches for virtual screening has been incentivized by the presence of numerous biological and chemo-structural information of natural products deposited in public databases (Valli et al., 2013; Harvey et al., 2015; Pilon et al., 2017), as well as by the advances of computer processing and storage capacity (Walters, 2019). High scientific efforts to isolate and characterize natural products have increased the interest of the academia and industry to comprehensively organize this information using public databases to better explore these natural sources and also to contribute to our knowledge regarding their ethnobotanical information, biological activities, chemical structures, natural origin, and physicochemical properties. Herein, we do not intend to provide exhaustive information regarding these online databases with public access, but we will exhibit those with potential applications in virtual screening strategies of natural products.

### Nuclei of Bioassays, Ecophysiology, and Biosynthesis of Natural Products Database (NuBBE_DB_)

NuBBE_DB_ (https://nubbe.iq.unesp.br/portal/nubbe-search.html) provides information regarding chemo-structures obtained from Brazilian biodiversity (Valli et al., 2013). Currently, the database contains more than 2,200 structures of natural compounds obtained from different Brazilian biomes (Pilon et al., 2017). NuBBE_DB_ contains the 3D structures of natural products in an MOL2 file format, which is compatible with the most widely used molecular modeling and cheminformatics programs.

### Comprehensive Marine Natural Products Database (CMNPD)

The Comprehensive Marine Natural Products Database (CMNPD) (https://www.cmnpd.org/) is a comprehensive and curated marine natural products database that contains more than 32,000 structures (accessed on January 06, 2020) with different physicochemical and pharmacokinetic properties. Besides, it includes information regarding their biological activity, natural origin, and the geographical distribution of source organisms (Lyu et al., 2020). The database also contains the complete molecule datasets freely available for download (https://docs.cmnpd.org/downloads).

### Natural Product-Likeness Software Suite and Database (NaPLeS)

The natural product-likeness software suite NaPLeS (https://naples.naturalproducts.net/) is an MySQL database of natural products and an open-source web application that computes the natural product-likeness scores of large chemical libraries. Currently, the database contains 315,916 natural products from various public databases (Sorokina and Steinbeck, 2019).

### Universal Natural Product Database (UNaProd)

The Universal Natural Product Database (UNaProd) (http://jafarilab.com/unaprod/index.php) is an online and public database of natural products used in Iranian traditional medicine. The database currently contains 2,696 compounds of botanical, animal, and mineral origins (accessed on January 06, 2020) (Naghizadeh et al., 2020).

### Natural Product Activity and Species Source Database (NPASS)

The Natural Product Activity and Species Source Database (NPASS) (http://bidd.group/NPASS/index.php) provides biological activity results and information regarding the origin species of more than 35,032 natural products (accessed on January 06, 2020) (Zeng et al., 2018). The database also contains a structural compound library freely available for download in SDF and SMILES formats (http://bidd.group/NPASS/downloadnpass.html).

### BIOFACQUIM

BIOFACQUIM (https://biofacquim.herokuapp.com/) is a free and public database of natural products isolated and characterized from Mexican biodiversity. Compounds from this database are also available in the ZINC database (Pilón-Jiménez et al., 2019). Currently, the database contains 423 natural compounds (accessed on January 08, 2020) which are identified by their respective names, accession codes, source organisms, in SMILE format, and references.

### Natural Products Atlas

The Natural Products Atlas (https://www.npatlas.org/joomla/) is an open-access database of microbial natural products that contain 24,594 compound structures (accessed on January 07, 2020) and information related to their structure, IUPAC name, source organisms, and literature (van Santen et al., 2019). The database also contains information of other natural product databases, such as the Minimum Information about a Biosynthetic Gene Cluster (MIBiG) repository and the Global Natural Products Social Molecular Networking (GNPS) platform (van Santen et al., 2019).

### African Natural Products Database (ANPDB)

The African Natural Products database (ANPDB) is a free database of natural products from different regions of the African continent (available at ANPDB|ANPDB (African-compounds.org) and contains ~4,500 structures (accessed on January 12, 2020). The available data content comprises sources covering the period from 1962 to 2019 (Ntie-Kang et al., 2017). The database also contains the 3D structures of natural products in SMILES and SDF formats available for non-commercial uses.

### Natural Products for Cancer Regulation (NPCARE)

The Natural Products for Cancer Regulation (NPCARE) is a free online database (http://silver.sejong.ac.kr/npcare/) that provides more than 6,000 natural products and more than 2,000 extracts isolated from 1,952 different species including microorganisms, marine organisms, and plants, as well as information related to the action of these extracts and isolated natural compounds against the gene expression levels and cancer cell line inhibition (Choi et al., 2017). The database is an interesting source to discover potential anticancer compounds and to understand the anticancer molecular mechanisms underlying natural products.

### StreptomeDB 3.0

StreptomeDB (http://www.pharmbioinf.uni-freiburg.de/streptomedb) is a free and online database used to explore natural products isolated or mutasynthesized from streptomycetes using an interactive phylogenetic analysis (Lucas et al., 2013; Moumbock et al., 2021). StreptomeDB 3.0 provides more than 6,500 natural products obtained from ~3,300 *Streptomyces* strains (Moumbock et al., 2021). These metabolites show interesting biological activities, such as antimicrobial, anticancer, and immunosuppressant properties. The compound structures are identified by their respective source organisms, references, biological role, and the routes of biosynthesis.

## Final Considerations

Natural products offer an interesting structural scaffold, helping to find new chemical entities with several industrial applications, thus offering innovative solutions to solve old worldwide problems, such as bacterial resistance against antibiotics (Smith et al., 2018; Newman and Cragg, 2020). However, the complex and highly diverse structure and the peculiar chemical space occupied by natural products have imposed pharmacokinetic and pharmacodynamic limitations, thus restricting their use for specific purposes by the pharmaceutical and cosmetic industries.

Several computational methods applied in virtual screening strategies have been developed over the years, thus increasing the rational explorations of natural sources aiming at the identification of specific bioactive compounds from large chemo-structural libraries. These computational strategies have also opened up new opportunities to discover new industrial applications of natural compounds justifying the financial and time efforts for their exploration. Natural products present a high structural diversity when compared with their synthetic counterparts, and their difference is, in part, due to the existing intricate biosynthetic pathways in living organisms that produce derived structures, containing modified functional groups, such as glycosylation and methylation. Based on these, the virtual screening strategies must investigate the chemical space of natural products, seeking to identify some classes of compounds with bioactivity or structural scaffolds present in known active molecules. Some of these screening strategies include applying less restrictive structural-based similarity cutoffs (Pavadai et al., 2017) and the modeling of hypothetically derived natural product structures (Skinnider et al., 2017). Regarding the application of molecular filters, some “bioactivity-likeness” criteria must be used with caution to avoid misleading screening or remotion of the important structural diversity of the compound libraries since the structural complexity of natural products situates them beyond the acceptable limits of some empirical rules determined by these filters.

Artificial intelligence algorithms employed in ligand-based approaches have demonstrated high success rates in finding interesting compounds with reduced computational time, and their combined uses with cheminformatics and molecular modeling methods have increased the efficiency of virtual screening strategies, allowing us to explore the highly diverse chemo-structural landscapes of natural products.

Here, we hope to encourage the use of these computational tools by experimental groups, helping researchers to familiarize themselves with their concepts and capabilities as well as alert them of some of the common biases faced by investigators during the investigation of natural sources using computational tools, citing some possible solutions. Finally, we indicate that the automatic process represented by virtual screening must be oriented by human expert decision to avoid misinterpretation or false findings, and also to select compounds based on their desirable features, such as commercial availability, low cost, and synthetic feasibility.

## Author Contributions

KS, RB, and JL: conceptualization. KS, LN, AL, and VD: investigation. KS, LN, VD, and RB: writing—original draft preparation. KS, AL, CN, RB, and JL: writing—review and editing. CN, RB, and JL: supervision. All authors have read and agreed to the published version of the manuscript.

## Conflict of Interest

RB was employed by company InsilicAll Ltda. The remaining authors declare that the research was conducted in the absence of any commercial or financial relationships that could be construed as a potential conflict of interest.
